# The SeqFEATURE library of 3D functional site models: comparison to existing methods and applications to protein function annotation

**DOI:** 10.1186/gb-2008-9-1-r8

**Published:** 2008-01-16

**Authors:** Shirley Wu, Mike P Liang, Russ B Altman

**Affiliations:** 1 Program in Biomedical Informatics, Stanford University, Stanford, CA, 94305 USA; 2 Google, Inc., Amphitheatre Pkwy, Mountain View, CA, 94043 USA; 3 Department of Genetics, Stanford University, Stanford, CA, 94305 USA; 4 Department of Bioengineering, Stanford University, Stanford, CA, 94305 USA

## Abstract

Structural genomics efforts have led to increasing numbers of novel, uncharacterized protein structures with low sequence identity to known proteins, resulting in a growing need for structure-based function recognition tools. Our method, SeqFEATURE, robustly models protein functions described by sequence motifs using a structural representation. We built a library of models that shows good performance compared to other methods. In particular, SeqFEATURE demonstrates significant improvement over other methods when sequence and structural similarity are low.

## Abstract 2

SeqFEATURE, a tool for protein function annotation, models protein functions described by sequence motifs using a structural representation. The tool shows significantly improved performance over other methods when sequence and structural similarity are low.

## Background

With the complete genomes sequenced for an increasing number of organisms, emphasis is shifting from identifying genes and gene products to understanding protein function and the interactions between biological entities on a systems level. Molecular-level descriptions of cellular physiology are critical for elucidating biological processes and manipulating them for medical or industrial purposes, such as bioremediation or drug design. In particular, the three-dimensional structures of proteinsprovide clues about their functions and how function may be manipulated by mutation or with small molecule chemicals. With protein structure determination becoming more efficient, the number of available structures is growing rapidly. The emergence of structural genomics [1], which aims to solve a representative set of proteins covering the entire space of naturally occurring structural folds, has spurred this growth, and depositions in the Protein Data Bank (PDB) [2] from structural genomics projects accounted for 16% of new structures in 2006, almost double the percentage in 2003 [3]. Structural genomics data and targets from almost all structural genomics centers are stored centrally in TargetDB [4], a database accessible from the PDB.

Because a major goal of structural genomics is to sample the entire protein structure space, many of the structural genomics centers target proteins with novel folds and low sequence identity to known proteins. The number of structures released per year by structural genomics has grown to almost 1,400 in 2007, with about 50% of these having less than 30% sequence identity to the rest of the PDB [3]. The consequence of this growth is that many of the new structures being deposited into the PDB lack functional annotation. Learning the functions of these new proteins will enable us to take best advantage of structural genomics efforts, but using conventional experimental methods can be tremendously time-consuming and expensive without testable hypotheses. As the field of structural genomics matures, the bottleneck from structure determination to functional annotation will become more pronounced. Automated function prediction programs would greatly alleviate this problem by providing clues and focusing investigation.

Many function prediction programs exist, ranging from those that describe general physicochemical properties of proteins to those that characterize functional domains or predict specific enzymatic activity. The majority of predictors use primary sequence, and the simplest method is to use a sequence alignment algorithm such as BLAST [5], since high sequence similarity is almost always indicative of evolutionary - and, often times, functional - conservation. Wilson *et al*. [6] showed that precise function can be transferred reliably above 40% and broad functional class above 25% sequence identity. In addition, many tools take advantage of curated databases, such as the manually inspected profile-Hidden Markov Models (HMMs) contained in the Pfam database of protein families [7], and PROSITE, which consists of manually built sequence patterns and profiles [8].

Both Pfam and PROSITE are contained within InterPro [9,10], a comprehensive, integrated resource for protein sequence information that provides many databases and tools for protein function and domain recognition. Among the tools offered are other HMM-based methods [11] such as HMMTigr, built on the TIGRFAMs database [12], and HMMPanther, built on the PANTHER database [13], both of which focus on function-based classification. Superfamily [14], another HMM-based tool hosted on InterPro, classifies sequences using manually curated models built from the Structural Classification of Proteins (SCOP) [15]. As a complement to Superfamily, Gene3D [16] is a semi-manually curated set of models built using the CATH protein structure classification [17].

Sequence-based tools often provide useful information about function, but they may be less well suited to cases where sequence identity is low. Under these circumstances, structure-based tools may detect functional signals that sequence-based methods are unable to capture due to sequence divergence. Since a protein's structure and function are inexorably linked, structure-based tools can abstract out those elements that are necessary for defining a particular function independent of the linear sequence, lending a degree of sensitivity and specificity that may improve over sequence-based tools. The abstractions can range in scale from entire secondary structure elements to residue or atom-based features. Function annotation based on structure is usually limited to recognition of either general folds or low-level molecular functions such as binding sites and active sites; it is unlikely routinely to predict the overall biological pathways and processes in which a protein participates. However, a complete understanding of structural environments and binding and active site properties provides a pyramid of evidence for the functional roles of a protein.

A number of structure-based function prediction methods have been developed to take advantage of the surge of new protein structures. Some of these rely on expert knowledge for defining the features useful for classifying a particular functional site, while others learn the important features through supervised machine learning approaches. An example of the former is Fuzzy Functional Forms [18], which are three-dimensional descriptions of functional sites based on conserved geometry, protein conformation, and residue identity. The descriptions are built by hand using information from solved crystal structures and published literature, and were able to help identify functional sites in structures whose sequence similarity to known proteins was low enough to render sequence-based tools ineffective [19].

Constructing models manually is time-consuming, however, and several more tractable methods have since been developed. ProKnow [20] uses features extracted from sequence or structure via established tools such PSI-BLAST [21], DALI [22], PROSITE, and the Database of Interacting Proteins (DIP) [23] to map proteins to functional terms in the Gene Ontology (GO) [24]. An alternative method by Polacco and Babbitt [25], called Genetic Algorithm Search for Patterns in Structures, or GASPS, constructs short three-dimensional motifs of functional sites consisting of conserved residues through an iterative mutation and selection process. Secondary Structure Matching (SSM) uses a graph-based representation of secondary structure to find similar structural matches to a query structure from the PDB [26]. Laskwoski *et al*. [27] presented the idea of 3D templates, which are spatial arrangements of three residues representative of functional sites or ligand-binding sites. These can be built from known examples and matched to the query, or the query structure itself can be broken into 'reverse' templates and matched against the PDB.

Perhaps the most ambitious solution to the problem of automated function prediction is ProFunc [28]. Combining about a dozen different sequence and structure-based methods, including database pattern searches, SSM, and 3D templates, ProFunc offers an impressively complete arsenal of methods for function prediction in one convenient, web-based tool. A recent study tested ProFunc's usefulness in predicting function for structural genomics targets and found that SSM and 3D templates were most effective [29]. To determine correctness of their predictions, they compared GO terms between the query and potential hit.

One difficulty with evaluating the performance of function prediction methods is the complex way in which protein function is defined. Function commonly describes specific enzymatic activities such as isomerization or phosphorylation, but it also encompasses binding to macromolecules or cofactors, modification sites for the attachment of lipids or other molecules, and general association in a biological pathway or complex. Although there are many classification schemes that cover one or more types of function, there is no functional classification describing all types of function that allows comparisons between different levels of the classification. The Enzyme Commission (EC) system [30] is widely accepted for enzyme classification, but it does not describe non-enzymatic functions or take into account sequence conservation or mechanism, which can indicate an evolutionary relationship [31]. The GO database is comprehensive and its terms are extremely popular for biological annotation, but it is difficult to compare terms when function predictions are made at different levels of the GO hierarchy.

The outputs of function prediction methods are also difficult to compare; for example, SSM returns an entire structure or portion of a structure that matched the query, while 3D templates returns either a precise prediction of function at a three-dimensional location in the query, or a protein containing similar residue geometry to the query, depending on the type of template chosen. The ambiguity behind the concept of 'function' and even its location in a structure with the concomitant diversity of frameworks and outputs make it very challenging to compare the performance of different methods. By restricting a comparison to a subset of functions that is relatively well defined, however, such as enzymatic functions, one can gain an impression of how each method performs.

Here, we present and apply SeqFEATURE, an automated method for protein function annotation from structure that is an extension of FEATURE, a more general framework published previously [32]. FEATURE models the local three-dimensional microenvironment surrounding functional sites and is, therefore, mostly independent of sequence or structure homology. Although FEATURE performs well [33], the need for manually curated sets of positive training examples may limit its utility. SeqFEATURE addresses this limitation by automatically extracting training sets from the PDB using sequence motifs as seeds [34]. The FEATURE framework models three-dimensional motifs using physical and chemical properties, and thus attempts to generalize the one-dimensional motif by recognizing similar three-dimensional environments that do not share significant one-dimensional similarity. We have used SeqFEATURE to build a library of functional site models from PROSITE motifs and evaluated its performance through cross-validation. Importantly, we also compared SeqFEATURE to PROSITE, Pfam, HMMPanther, Gene3D, SSM and 3D templates, and further examined each method's performance in low sequence identity and low structural similarity situations.

As a first step in aiding structural genomics and function prediction efforts, we have applied SeqFEATURE in a comprehensive scan of the entire PDB and focused our analysis on structures from the TargetDB repository of structural genomics targets. We report several interesting case studies from this analysis and compare SeqFEATURE's predictions to those of other methods. All data from the scan and all of the functional site models created to date are available for download [35]. Additionally, one can scan any structure in PDB format with the full library of SeqFEATURE models.

## Results

We built a library of 3D functional site models using the FEATURE algorithm applied to training sets extracted automatically through sequence motifs found in the PDB. The library was evaluated using cross-validation and compared to existing sequence and structure-based function prediction methods. We also investigated potential functions for structures with unknown function.

### SeqFEATURE model library

**Figure 1:**
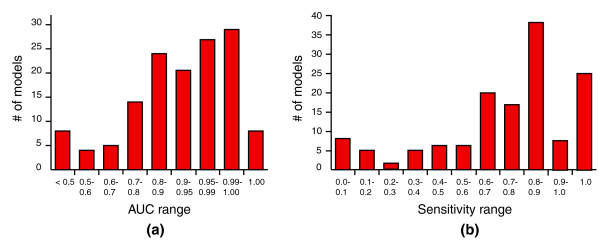
Distribution of AUC and sensitivity for all SeqFEATURE models listed in Table 2. **(a) **Distribution of model AUC. Most models have AUC greater than 0.8, with 47% having AUC >0.95 and a few poor performers less than 0.5. **(b) **Distribution of model sensitivity. We plot the sensitivity of each model at the default score cutoff of 99% specificity based on training data. Most models have a sensitivity greater than 0.6-0.7 at this cutoff, and many have a sensitivity greater than 0.8.

The SeqFEATURE library consists of 136 models derived from 53 PROSITE patterns (Table 1). Of these models, 105 (77%) have an AUC greater than 0.8, and 64 (47%) have an area under the curve (AUC) greater than 0.95 (Figure 1a). Sensitivity at the default 99% specificity cutoff is slightly more variable, but 82% of the models have sensitivity greater than 0.5 and 59% have sensitivity greater than 0.75 (Figure 1b).

**Table 1:** SeqFEATURE models built from PROSITE motifs

PROSITE pattern	Position(s)	Residue	Atom(s)
2FE2S_FERREDOXIN	1, 6, 9	Cys	SG
4FE4S_FERREDOXIN	1, 3, 5, 7	Cys	SG
AA_TRANSFER_CLASS_1	4	Lys	NZ
AA_TRANSFER_CLASS_2	4	Lys	NZ
AA_TRANSFER_CLASS_3	19	Lys	NZ
ADH_SHORT	3	Tyr	OH
ADH_ZINC	2	His	ND1, NE2
ADX	6, 9	Cys	SG
ALDEHYDE_DEHYDR_CYS	6	Cys	SG
ALDEHYDE_DEHYDR_GLU	2	Glu	OE1, OE2
ASP_PROTEASE	4	Asp	OD1, OD2
ASX_HYDROXYL	3	Asn	ND2, OD1
BETA_LACTAMASE_A	5	Ser	OG
BETA_LACTAMASE_B_1	4, 6	His	ND1, NE2
	8	Asp	OD1, OD2
BPTI_KUNITZ_1	4, 8	Cys	SG
C_TYPE_LECTIN_1	1	Cys	SG
CARBOXYLESTERASE_B_1	11	Ser	OG
CARBOXYLESTERASE_B_2	3	Cys	SG
CHITINASE_18	9	Glu	OE1, OE2
COPPER_BLUE	11	His	ND1, NE2
	7	Cys	SG
CYTOCHROME_P450	8	Cys	SG
EF_HAND	1, 3, 5, 9	Asp	OD1, OD2
	7, 12	Tyr	OH
	3, 5, 9	Asn	ND2, OD1
	5, 9	Ser	OG
	7, 9	Thr	OG1
	7	Glu	OE1, OE2
	7	Lys	NZ
EGF_1	1, 3, 7	Cys	SG
EGF_2	1, 3, 8	Cys	SG
GLYCOSYL_HYDROL_F10	7	Glu	OE1, OE2
GLYCOSYL_HYDROL_F5	7	Glu	OE1, OE2
HIPIP	1, 7	Cys	SG
HMA_1	5, 8	Cys	SG
IG_MHC	3	Cys	SG
IMP_1	4	Asp	OD1, OD2
KAZAL	1, 3, 7, 9	Cys	SG
LIPASE_SER	7	Ser	OG
LIPOYL	9	Lys	NZ
PA2_HIS	4	His	ND1, NE2
PEROXIDASE_1	8	His	ND1, NE2
PEROXIDASE_2	8	His	ND1, NE2
PHOSPHOPANTETHEINE	6	Ser	OG
PROTEIN_KINASE_ST	5	Asp	OD1, OD2
PTS_HPR_SER	5	Ser	OG
RNASE_T2_1	4	His	ND1, NE2
SHIGA_RICIN	5	Glu	OE1, OE2
	8	Arg	NE, NH1, NH2
SMALL_CYTOKINES_CC	1, 2, 11, 17	Cys	SG
SNAKE_TOXIN	2, 4, 7, 8	Cys	SG
SUBTILASE_ASP	5	Asp	OD1, OD2
THIOL_PROTEASE_ASN	6	Asn	ND2, OD1
THIOL_PROTEASE_HIS	3	His	ND1, NE2
THIOREDOXIN	8, 11	Cys	SG
TRYPSIN_HIS	5	His	ND1, NE2
TRYPSIN_SER	6	Ser	OG
TYR_PHOSPHATASE_1	3	Cys	SG
UBIQUITIN_CONJUGAT_1	10	Cys	SG
ZINC_FINGER_C2H2_1	1, 3	Cys	SG
	7, 9	His	ND1, NE2
ZINC_PROTEASE	3, 7	His	ND1, NE2
	4	Glu	OE1, OE2

Some PROSITE patterns have multiple functional residues, and so more than one model was built for these patterns (for example, multiple EF_HAND models are built). Many hits will score high on all models, but distantly related sites may hit only a subset. SeqFEATURE models are named by concatenating the PROSITE-PATTERN, POSITION, RESIDUE and ATOM for unambiguous identification.

**Figure 2:**
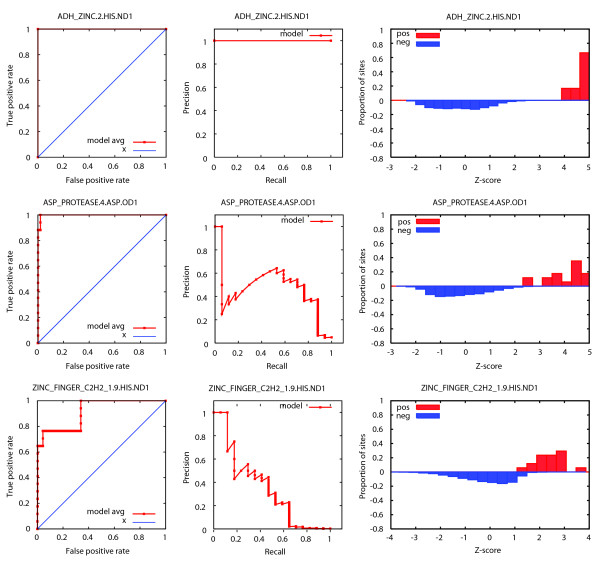
Example ROC curves, precision-recall curves, and Z-score distributions for SeqFEATURE models. A sample of performance plots for ADH_ZINC.2.HIS.ND1 (top), ASP_PROTEASE.4.ASP.OD1 (middle), and ZINC_FINGER_C2H2_1.9.HIS.ND1 (bottom) are shown, representing a model with excellent performance, good performance, and somewhat satisfactory performance, respectively. The leftmost plot in each row gives the ROC curve in red and random performance in blue, the middle plot shows the precision versus recall (sensitivity) curve, and the rightmost plot shows the distribution of scores for positive sites (red) and negative sites (blue) from training. Because there are many more negative sites than positive sites, the score distributions on the right are normalized to Z-scores.

Receiver operating characteristic (ROC) curves from cross-validation and Z-score distributions of the final models can be used together to evaluate the ability of the model to distinguish true sites from the background. We evaluate the separation between the positive and negative sites by plotting the distributions of Z-scores for the positive and negative training examples. Plots of positive predictive value (PPV) versus sensitivity give the proportion of total hits to the models that are true positives as a function of sensitivity. Representative examples of ROC curves, PPV versus sensitivity curves, and Z-score distributions for a range of model performances are shown in Figure 2.

Table 2 lists the top 25 performing SeqFEATURE models ranked by AUC. The sensitivity of these models is, in general, very high, especially at the default 99% specificity Z-score cutoffs. Even at 100% specificity over half of the models have greater than 0.75 sensitivity. This list also contains a wide range of PROSITE patterns, indicating that the method performs very well for many different types of functional sites.

**Table 2:** Top 25 performing SeqFEATURE models

Model name	AUC	Z-cutoff-100	Sens-100	Z-cutoff-99	Sens-99	Z-cutoff-95	Sens-95	No. of examples
ADH_ZINC.2.HIS.ND1	1.0000	3.4172	1.0000	2.4184	1.0000	1.7128	1.0000	6
CARBOXYLESTERASE_B_1.11.SER.OG	1.0000	5.2823	1.0000	2.5415	1.0000	1.7047	1.0000	6
GLYCOSYL_HYDROL_F5.7.GLU.OE1	1.0000	3.8385	1.0000	2.7188	1.0000	1.8787	1.0000	6
HIPIP.7.CYS.SG	1.0000	4.8473	1.0000	2.7401	1.0000	1.7795	1.0000	5
RNASE_T2_1.4.HIS.NE2	1.0000	4.0910	1.0000	2.6209	1.0000	1.6521	1.0000	5
THIOL_PROTEASE_ASN.6.ASN.ND2	1.0000	3.8379	1.0000	2.5982	1.0000	1.8237	1.0000	5
THIOL_PROTEASE_ASN.6.ASN.OD1	1.0000	4.1252	1.0000	2.6255	1.0000	1.7985	1.0000	5
TYR_PHOSPHATASE_1.3.CYS.SG	1.0000	5.4246	1.0000	2.7473	1.0000	1.7596	1.0000	8
CYTOCHROME_P450.8.CYS.SG	1.0000	4.1254	0.8333	2.4019	1.0000	1.7526	1.0000	12
PEROXIDASE_2.8.HIS.ND1	0.9999	3.6628	0.8000	2.6225	1.0000	1.7515	1.0000	5
BPTI_KUNITZ_1.8.CYS.SG	0.9999	3.5059	0.8333	2.2843	1.0000	1.7820	1.0000	6
4FE4S_FERREDOXIN.5.CYS.SG	0.9999	2.7500	0.4000	1.5623	1.0000	1.2660	1.0000	17
ADH_SHORT.3.TYR.OH	0.9999	5.0745	0.1176	2.2891	1.0000	1.6249	1.0000	20
TRYPSIN_SER.6.SER.OG	0.9998	5.4646	0.0000	2.1696	1.0000	1.6085	1.0000	17
GLYCOSYL_HYDROL_F5.7.GLU.OE2	0.9998	3.9203	0.8333	2.6280	1.0000	1.8757	1.0000	6
4FE4S_FERREDOXIN.1.CYS.SG	0.9998	3.1084	0.1000	2.0501	1.0000	1.5589	1.0000	20
PEROXIDASE_2.8.HIS.NE2	0.9997	3.9233	0.6000	2.5753	1.0000	1.8144	1.0000	5
BETA_LACTAMASE_B_1.6.HIS.ND1	0.9997	5.3466	0.8000	2.7205	1.0000	1.7821	1.0000	5
ADH_ZINC.2.HIS.NE2	0.9996	3.8970	0.6667	2.4840	1.0000	1.7499	1.0000	6
LIPASE_SER.7.SER.OG	0.9995	4.7293	0.6250	2.5387	1.0000	1.7350	1.0000	8
ASP_PROTEASE.4.ASP.OD2	0.9994	3.7837	0.4706	2.2973	1.0000	1.7238	1.0000	17
IMP_1.4.ASP.OD2	0.9994	3.9608	0.6000	2.5508	1.0000	1.8129	1.0000	5
BETA_LACTAMASE_B_1.4.HIS.ND1	0.9993	3.8683	0.8000	2.6032	1.0000	1.8239	1.0000	5
BETA_LACTAMASE_B_1.8.ASP.OD1	0.9991	4.6363	0.6000	2.9202	1.0000	1.8558	1.0000	5
4FE4S_FERREDOXIN.3.CYS.SG	0.9991	3.4191	0.0000	2.0044	0.9500	1.4919	1.0000	20

For each model, we report (from left to right) the AUC, the Z-score for which all negatives are correctly predicted, the sensitivity (Sens) at this Z-score, the Z-score for which 99% of negatives are correctly predicted, the associated sensitivity, and the Z-score and associated sensitivity for 95%. The final column reports the number of positive examples of the one-dimensional motif found in the ASTRAL40 compendium of the PDB.

### Methods comparison

Cross-validation is not necessarily representative of how a model will perform on independent test data. In order to get a more realistic estimate of the library's performance, we constructed a specialized test set from the PROSITE records for each pattern, which contain manually curated annotations of true positives, false positives, and false negatives. The test sets consisted, therefore, of structures that the associated PROSITE pattern is known to detect correctly, falsely predict, and altogether miss.

**Figure 3:**
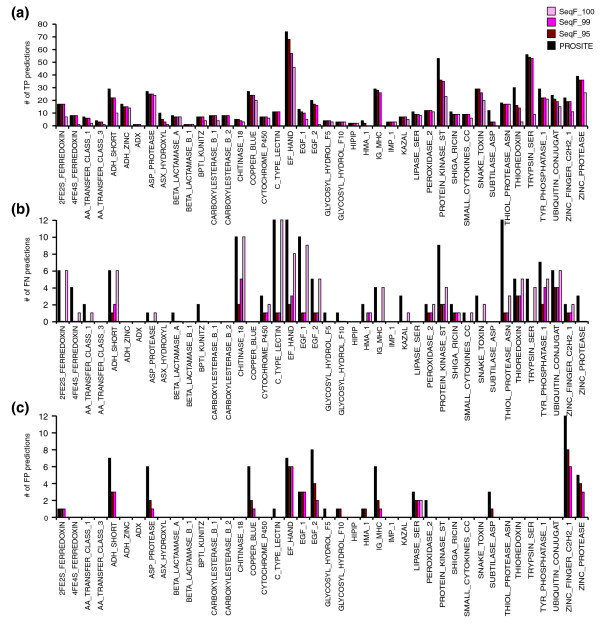
Performance on PROSITE true positives, false positives, and false negative test sites. We show the **(a) **true positive (TP), **(b) **false negative (FN), and **(c) **false positive (FP) prediction rates for SeqFEATURE (at 95%, 99%, and 100% specificity) and PROSITE on test sites derived from the corresponding PROSITE patterns. The PROSITE values represent the maximum possible for each category. Not all patterns had a false negative or false positive test set. Most of SeqFEATURE's incorrect predictions at 95% and 99% specificity cutoffs arise from poor performance on a small subset of the patterns.

**Figure 4:**
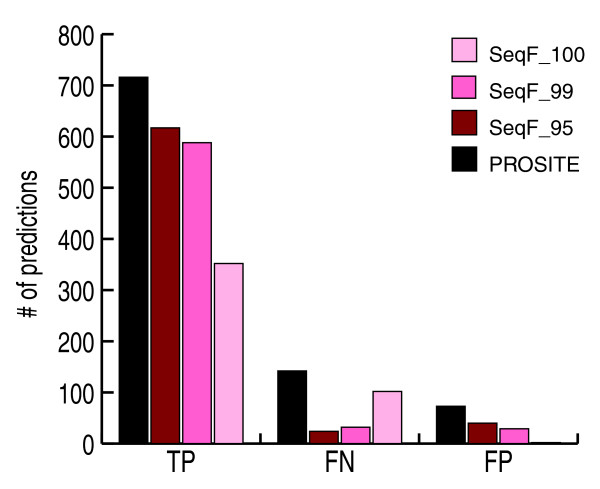
Summary performance on PROSITE true positives (TP), false positives (FP), and false negative (FN) test sites. We summarize total numbers of predicted true positives, false negatives, and false positives for PROSITE and SeqFEATURE at 100%, 99%, and 95% specificity cutoffs. SeqFEATURE (at the default 99% specificity cutoff) misses about 18% of the PROSITE true positives on average, but it also predicts 60% fewer false positives and 78% fewer false negatives than PROSITE. The three different specificity cutoffs also show tradeoffs in the numbers of true positives and false predictions made by SeqFEATURE, demonstrating that one can adjust the cutoff to fit desired performance. For example, one can attain a very high positive predictive value by using SeqFEATURE's 100% specificity cutoffs - although sensitivity decreases to about 50%, almost no false positive predictions are made.

Importantly, we could directly compare if and where SeqFEATURE outperforms the originating PROSITE pattern. Figure 3a-c show the numbers of true positives, false negatives, and false positives predicted by SeqFEATURE at varying specificity-based score cutoffs compared to the corresponding PROSITE pattern. Figure 4 shows overall numbers of predictions in each category. Since the test sets were derived from PROSITE, the PROSITE values represent the maximum that could possibly be obtained for each type of prediction. While SeqFEATURE does not predict all true positives correctly, it predicts 82% of true positives correctly at the default 99% specificity cutoff. At the same cutoff, SeqFEATURE also predicts about 78% fewer false negatives than PROSITE, and about 60% fewer false positives.

When we compared performance between SeqFEATURE, Pfam, HMMPanther, and Gene3D (restricting the comparison to the high confidence assigned patterns for each sequence-based method as described in Materials and methods), we found Gene3D to be the best performing method by far, with sensitivity just over 98%, specificity at 85.4%, and PPV at 99% (Table 3). Pfam was the second most sensitive method at 93.7%; since it predicted all negative examples (PROSITE false positives) correctly, Pfam had a PPV of 100%. HMMPanther scored slightly below Pfam on its limited test set with a sensitivity of 91.9%; there were not enough examples to evaluate specificity. SeqFEATURE had a sensitivity of 86.2% at our most lenient cutoff, and specificity and PPV comparable to Pfam and Gene3D at our more stringent cutoffs. Interestingly, all of the sequence-based methods show a marked decrease in sensitivity when evaluated only on positive examples that did not contain the PROSITE motif (that is, PROSITE false negatives). SeqFEATURE, on the other hand, is not as significantly affected by whether the test proteins contain the canonical sequence motifs.

**Table 3:** Comparison of SeqFEATURE (at three specificity-based score cutoffs) to Gene3D, Pfam, and HMMPanther (Panther)

	Gene3D	Pfam	Panther	SeqF_95	SeqF_99	SeqF_100
True positive sensitivity	**0.998**	**0.937**	**0.919**	0.862	0.821	0.492
False negative sensitivity	**0.907**	0.704	0.532	**0.831**	**0.775**	0.282
Overall (TP + FN) sensitivity	**0.983**	**0.898**	0.831	**0.857**	0.814	0.457
(False positive) specificity	**0.854**	**1.000**	-	0.452	0.603	**0.973**
Positive predictive value	**0.990**	**1.000**	-	0.948	0.960	**0.995**
Sensitivity at <35% seq-ID	**0.925**	**0.761**	0.639	**0.769**	0.699	0.316
Sensitivity at <30% seq-ID	**0.869**	0.738	0.618	**0.869**	**0.783**	0.400
Sensitivity at <25% seq-ID	**0.600**	0.467	0.250	**0.933**	**0.786**	0.429

We calculated the sensitivity (on PROSITE true positives (TP), PROSITE false negatives (FN), and overall), specificity, PPV, and sensitivity at varying levels of sequence identity for each method using the portion of the test set corresponding to the coherent patterns for that method. Bold values indicate the top three performing methods for that row. The sequence-based methods are expected to do very well since they have the advantage of abundant sequence data for modeling functional families. SeqFEATURE performs relatively less well in sensitivity and specificity, but is still very competitive, especially with regards to positive predictive value and false negative sensitivity. At lower sequence identities, Gene3D outperforms all other methods at sequence identities greater than 30%, but SeqFEATURE remains robust throughout and, in particular, shows better performance than Gene3D and Pfam when sequence identity is less than 30%. SeqFEATURE is the best performing method by far when the proteins have less than 25% sequence identity to the training set.

On the randomized sample test set (see Materials and methods), we were able to compare SeqFEATURE to 3D templates and SSM (Table 4). Here, SeqFEATURE's best sensitivity increased to 93%, though its best specificity dropped to 93%. PPV decreased slightly to 94% at the most stringent cutoff. 3D templates performed most well out of the structure-based methods, with 90% sensitivity, 100% specificity, and a PPV of 100%. SSM performed similarly to SeqFEATURE.

**Table 4:** Comparison of SeqFEATURE (at three specificity-based score cutoffs) to 3D templates and SSM

	3D templates	SSM	SeqF_95	SeqF_99	SeqF_100
Sensitivity	**0.897**	0.724	**0.931**	0.862	0.552
Specificity	**1.000**	**0.933**	0.600	0.667	**0.933**
PPV	**1.000**	**0.955**	0.818	0.833	0.941
LSS-sensitivity	0.200	0.267	**0.533**	**0.467**	0.133

We calculated the sensitivity, specificity, and PPV for SeqFEATURE, 3D templates, and SSM using a random subset of 29 positive and 15 negative structures. Additionally, we calculated sensitivity for each method on a low structural similarity subset ('LSS-sensitivity') of the positive test set. Bold values indicate the top two performing methods for that row. SeqFEATURE performs relatively less well overall, but when structural similarity is reduced, SeqFEATURE again is the best performing method.

**Figure 5:**
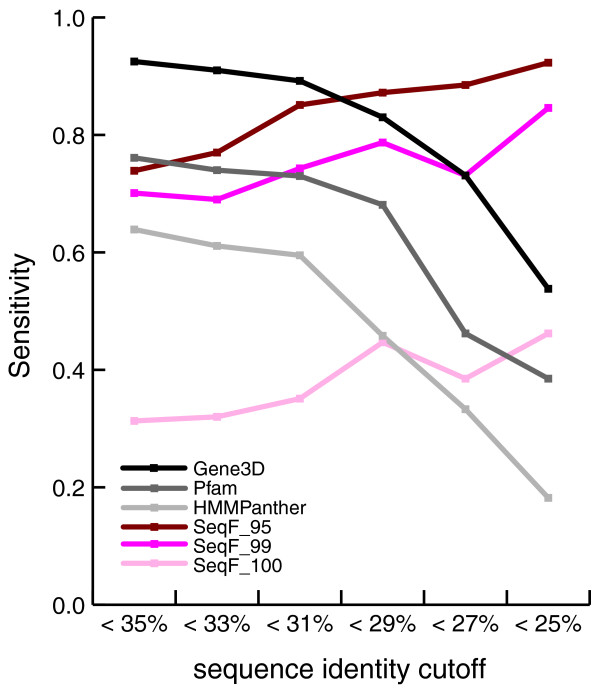
Sensitivity trends of SeqFEATURE, Gene3D, Pfam, and HMMPanther at low sequence identities. We compared the sensitivity of SeqFEATURE at three specificity cutoffs against the sensitivity of Gene3D, Pfam, and HMMPanther on test sets filtered for low sequence identity. We evaluated each method on the subset of the original test set that had less than the specified sequence identity to the training sets. As sequence identity decreases, the sequence-based methods show a clear trend towards lower sensitivity. In contrast, SeqFEATURE at all three cutoffs shows no such downward trend, indicating robust detection of function even when sequence identity is very low.

Importantly, however, since the goal of many function prediction methods, including SeqFEATURE, is to aid in annotation of solved structural genomics targets, we also compared SeqFEATURE to the sequence-based methods using low sequence identity test sets to mimic the situation in which a newly solved structure has low sequence identity to proteins of known function. Table 3 shows the sensitivities of PROSITE, Gene3D, Pfam, HMMPanther and SeqFEATURE at 95% and 99% specificity cutoffs for test sets filtered at 25%, 30%, and 35% sequence identity to the training set. The sequence-based methods perform less well, particularly on sequences filtered at 30% and 25% identity. In contrast, SeqFEATURE achieves a sensitivity of 92.3% at the most lenient cutoff and 84.6% at the moderate cutoff for sequences with <25% sequence identity to the training sets. As shown in Figure 5, the sensitivity of sequence-based methods decreases directly in proportion to sequence identity, whereas the sensitivity of SeqFEATURE at all three cutoffs shows no downward trend. The observation that SeqFEATURE's performance remains robust reflects the fact that SeqFEATURE's true positive predictions are concentrated at lower sequence identities, suggesting that SeqFEATURE may be especially valuable in this scenario.

To determine whether the degree of structural similarity affects how well different methods predict function, we also constructed a low structural similarity test set using Dali pairwise matching between members of the low sequence identity test set and the corresponding positive training sets. Although relatively small (15 examples), the low structural similarity test set allows us to approximate the situation of function prediction on novel folds. As illustrated in Table 4, SeqFEATURE performs better at the 95% and 99% specificity cutoffs than the other structure-based methods; its low structural similarity (LS)-sensitivity is 53% and 47%, respectively, while the LS-sensitivity values for SSM and 3D templates are both less than 30%.

### Predictions of function for structural genomics targets

As of November 2007, TargetDB contained about 5,250 targets with structures released in the PDB; of these, about 1,500 were labeled only with 'structural genomics', 'unknown function', or 'hypothetical protein' in the PDB file header. Using the criteria of model AUC > 0.8, maximum score of that model's negative training set, and minimum score of that model's positive training set, we found 35 potential functional sites. We added one more predicted functional site that did not quite satisfy the criteria but had several such hits for multiple models for the same function, resulting in a total of 36 high-confidence predictions. We compare our predictions to those of PROSITE, Pfam, Gene3D, HMMPanther, SSM and 3D templates for the same structures.

In examining these structures, we found that some of them, though labeled as 'unknown function', actually had some functional annotation and, thus, we could determine the plausibility of our prediction. For example, PDB structure 1XRI is described as a putative phosphatase, and had a high scoring hit for the TYR_PHOSPHATASE_1.3.CYS.SG model. All of the other methods also detected phosphatase activity. Another example is 2E72, described as a zinc-finger containing protein, which hit our ZINC_FINGER_C2H2_1.1.CYS.SG model and for which Pfam, Gene3D, HMMPanther, SSM, and 3D templates all predicted zinc finger motifs.

More interesting, however, are predictions for structures that fail to garner any predictions from PROSITE, Pfam, Gene3D, or HMMPanther. Table 5 presents three of our most intriguing cases. In all of these cases, only SeqFEATURE gives a high-confidence prediction, though 3D templates and SSM sometimes offer matches to putative functions or have low-confidence predictions. In contrast, the SeqFEATURE predictions have relatively high Z-scores compared to the training set distributions.

**Table 5:** Predicted function for three structures from TargetDB

PDB ID	SeqFEATURE model	AUC	Site	Z-score	Cutoff	Other predictions
2EJQ	ZINC_PROTEASE.4.GLU.OE1	0.892	GLU96:A	4.574	-0.074	3D templates: probable anthrax toxin lethal factor
2OGF	EF_HAND.9.THR.OG1	0.920	THR17:D	4.675	3.370	SSM: aminopeptidase (Z-score = 2.7)
2OX6	EF_HAND.9.ASN.OD1	0.863	ASN8:B	4.102	2.498	3D templates: probable Zn enzyme

For each target, we provide the PDB identifier, the SeqFEATURE models that best hit the target, the locations hit by those models, the Z-scores, the corresponding 100% specificity Z-score cutoff for the model, and the predictions made by PROSITE, Pfram, 3D templates, and SSM. TargetDB structure 2EJQ has a high scoring zinc protease site. 2OGF and 2OX6 both had high scoring hits for calcium binding. Out of the other function prediction methods, only 3D templates and SSM had any predictions; these, however, were lower confidence.

**Figure 6:**
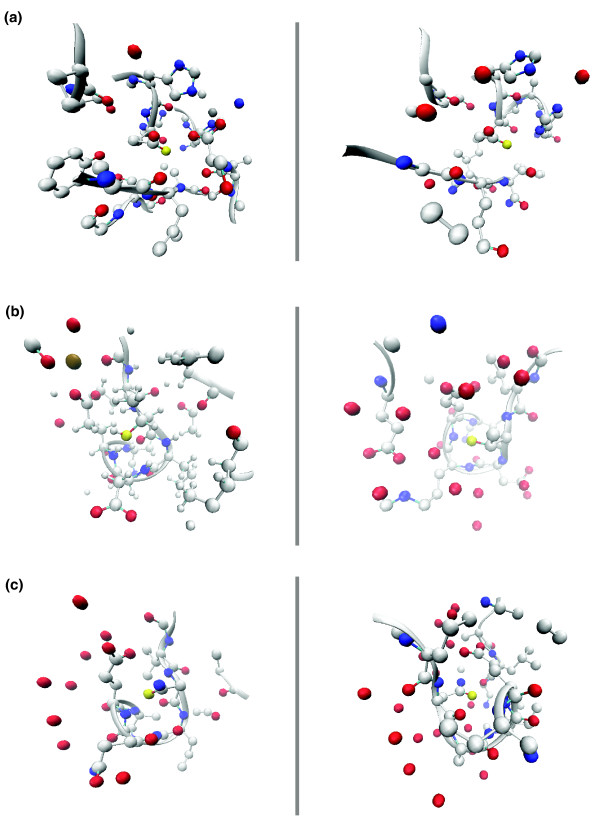
Local environments of SeqFEATURE predictions for three TargetDB structures with unknown function. Three predicted functional sites from TargetDB structures are shown compared to known examples of the predicted function. The predicted and known sites are shown in yellow, positively charge atoms (nitrogens) are shown in blue, and negatively charged atoms (oxygens) are shown in red. Carbons and secondary structure are shown in grey. All atoms within 7.5 angstroms of the site are shown. **(a) **The active site of a known zinc protease (1KAP) is shown to the left of a zinc protease site in 2EJQ predicted by SeqFEATURE. Note the presence of two histidine residues (one can be seen clearly above each site) and a number of negative charges distributed throughout both local environments. Note also the similarity in secondary structure. **(b) **The local structure of 1FI6 (left), which contains a known EF hand calcium-binding motif, is compared to SeqFEATURE's predicted calcium-binding site in 2OGF. Note the similar distribution of negative charges and closely matching loop structures. The calcium is visible as a brown sphere in 1FI6, surrounded by oxygen atoms. **(c) **1K8U is another known EF hand containing protein, shown to the left of the uncharacterized protein structure 2OX6, for which SeqFEATURE predicts calcium-binding. These figures were created using VMD [43].

The local environment surrounding high-confidence predicted sites in three TargetDB structures are shown in Figure 6 alongside positive training set examples of the corresponding SeqFEATURE model. Figure 6a shows 2EJQ, a conserved hypothetical protein from *Thermo thermophilus *to the right of 1KAP, a zinc metalloprotease from *Pseudomonas aeruginosa*. SeqFEATURE predicts an environment similar to that of a zinc protease, and, indeed, the two environments share the presence of two histidine residues and a number of negative charges in the vicinity of the site, as well as some common secondary structures. The other two cases are both predictions of calcium binding. 2OGF, an uncharacterized structure from *Methanococcus janaschii*, is compared to 1FI6, the calcium-binding domain of a protein involved in Ras signal transduction; 2OX6, an uncharacterized gene product from *Shewanella oneidensis*, is compared to 1K8U, a calcium regulatory protein. Both positive examples show an abundance of negative charges about five to seven angstroms from the motif residue. The predicted sites show comparable distributions of negative charges and contain loop structures similar to the positive examples. These three, in addition to SeqFEATURE's significant predictions for other TargetDB structures with unknown function, are especially interesting and warrant further study. All significant predictions for TargetDB structures are publicly available [36].

### Protein Data Bank scan

We additionally scanned every structure in the PDB - about 100 million potential sites - with every SeqFEATURE model. When we consider only those scores that came from models with an AUC of at least 0.95, and were greater than the 99% specificity cutoff defined for that model, 440,460 scores fit these criteria, or about 0.5% of the total number of scores generated. Filtering out redundant scores from proteins with multiple chains results in 298,870 predictions in 29,668 structures. The raw data from the scan are available for download [36]; further analysis of these predictions is beyond the scope of this paper. To access the full library scan for one structure, the user may query by PDB ID; alternatively, one can access all results by querying for a specific SeqFEATURE model.

## Discussion

SeqFEATURE extends earlier work on characterizing functional sites in protein structures by automating training set selection. We have used it to build a library of three-dimensional functional site models, 77% of which have an AUC greater than 0.8. When tested on untrained but known true positives, false positives, and false negatives from their corresponding PROSITE patterns, many models correctly classified all of the true positives and some of the false negatives, and had fewer false positive predictions than the pattern. Even when a model failed to recapitulate every PROSITE true positive, it often correctly predicted proteins that the PROSITE pattern missed.

Furthermore, we show that although SeqFEATURE demonstrates slightly lesser performance than the sequence-based methods overall, it exhibits useful performance trends as sequence identity to proteins of known function decreases. SeqFEATURE, and perhaps structure-based methods in general, should be most valuable in these scenarios, since they sense three-dimensional atomic environments rather than the sequences that fold to create those environments. We observe that this advantage is strongest when the sequence identity is less than 30%, which is well-documented as the 'twilight zone' of sequence analysis [37].

When we further investigate this region of low identity, we see that SSM and 3D templates do not perform as well as SeqFEATURE on the low structural similarity test set. SSM is essentially a fold-matching algorithm, and at low structural similarities the folds of the test structures likely differed significantly from those folds most representative of proteins with the function in question. Theoretically, the 3D template method is more similar to SeqFEATURE, but in reality it performed similarly to SSM. It is possible that the residue triads that 3D templates detect were dependent on exact conservation of sequence features. In contrast, SeqFEATURE was less affected by the reduction in structural similarity because it depends less on specific sequences or arrangements of residues, and instead incorporates abstract physical and chemical properties in a locally defined region.

Determining how different methods compare in predicting function is a challenging task, and so neither our procedure for comparing methods nor the interpretation of the comparison's results is straightforward. Function itself is broadly defined and does not lend itself easily to straightforward or computable classification schemes. Many classifications are applicable only to specific types of functions and can differ in the scope of their descriptions, ranging from whole domain labels on sequence (for example, Pfam) to exact locations in structures (for example, SeqFEATURE or 3D templates). Responding to this diversity in description and classification, we made several choices in our comparison of sequence and structure-based methods, each of which carries a certain amount of bias.

In comparing Pfam, HMMPanther, and Gene3D to other methods, for example, we restricted the evaluation to those functions (PROSITE patterns, specifically) whose SeqFEATURE positive training sets mapped unambiguously to the corresponding database assignment. This may have artificially boosted performance of the sequence-based methods, since we, in effect, considered only patterns with very high 'sensitivity' for each method to begin with based on our training sets. Interestingly, we also investigated HMMTIGR and Superfamily as other methods to include in the comparison, but these tools made very few predictions over the entire set of training and test structures, so we excluded them from the study.

Our choice of gold standard test sites from PROSITE may also be controversial because the test set is limited to those functional patterns that have been manually characterized and are thus subject to human judgment as well as human preference. In addition, due to the small number of test sites for most patterns, the results may be dominated by a few patterns with many test sites. Perhaps most obvious is the high probability that the negative test sites, by virtue of being defined as false positives with respect to the PROSITE pattern, are 'difficult cases'. This means that SeqFEATURE may be predisposed to low specificity, and specificity for all methods overall may suffer because the negative examples tend to be highly similar to the positive examples on at least the local sequence level.

The different types of input used to train each method also have some implications, an important one being that sequence-based methods currently have much more data available to them than structure-based ones. Although this means that the best sequence-based methods currently outperform structure-based methods on our unfiltered PROSITE-based test sets, it does not diminish the need for or value of structure-based methods. Such methods are useful precisely when sequence identity to known proteins is low, as shown in our results on low sequence identity test sets and our analyses on interesting TargetDB predictions.

The two structure-based methods compared here contain an analogous advantage, however, in that they match the query against the entire repository of known protein structures. Thus, if the query has very similar structures (for example, the same protein from different species) in the PDB, SSM and 3D template searches will very likely result in a high confidence hit to these structures. In cases where the query structure is completely novel, however, SSM and 3D templates are expected to do less well, as suggested by their performance on the low structural similarity test set. SeqFEATURE, on the other hand, because it does not rely on exactly conserved geometries or structural motifs, continues to show robust performance even when the structure does not share significant similarity to known proteins.

Another potential bias may come from limiting the structure-based comparisons to those patterns associated with EC numbers. In order to determine the correctness of predictions from SSM and 3D templates, we required a precise functional classification system. SCOP is a potential alternative evaluation method, but SCOP is a structural classification that does not always map directly to function, so we chose to use EC numbers. This, of course, means that the results of the comparisons may not be representative of how each method performs on non-enzymatic functions. The use of EC numbers is also affected by how accurately and completely the PDB is annotated and by the granularity of function assigned. Several of the test structures on which 3D templates and SSM performed poorly had matches to proteins annotated with only slightly different EC numbers. Thus, 3D templates and SSM should still be considered valuable tools for gaining insight into the potential function of an uncharacterized protein.

Although the set of patterns and the resulting test sets used here are by no means fully representative or without bias, they enabled us to map our SeqFEATURE models directly to test sets, a non-trivial endeavor given the inconsistency and variety of existing function classifications. It also allowed us to look specifically at where SeqFEATURE improves on or fares worse than the sequence patterns that generated the models. We often chose test sets with biases against our method in order to assess its operating characteristics accurately; for example, our use of one-dimensional sequence patterns as the gold standard provides a strong advantage to sequence-based methods. Restricting the comparison to patterns that mapped coherently to Pfam, Gene3D, and HMMPanther families may also predispose those methods to good performance. SeqFEATURE exhibited good performance despite these biases.

Because SeqFEATURE also focuses on the local microenvironment around functional sites, it can detect function at finer detail than fold-matching algorithms such as SSM. Because it considers both atom-based and physichochemical properties in addition to residue-based ones, it is also capable of generalizing function away from sequence and may be able to detect functional similarities that have converged from different ancestors or that use slightly different residues and a different overall fold to accomplish similar activities. This capability is demonstrated by the fact that SeqFEATURE detects many of the positive examples that the PROSITE pattern misses. The ability to abstract the properties relevant to function independent of sequence or structural homology is one of SeqFEATURE's biggest strengths.

Another one of SeqFEATURE's advantages is that score cutoffs can be adjusted to reflect the user's desired performance criteria, for example, estimated specificity, sensitivity, or positive predictive value. The ratio of true positives to false positives and false negatives is traded off depending on where the score cutoff is set. There are several additional filters one can use to boost the confidence of positive predictions. True hits often manifest themselves as a cluster of high-scoring positive predictions for the same or related functional site models. Single, isolated hits in a protein, although potentially interesting, may not have the exact function represented by the model.

**Figure 7:**
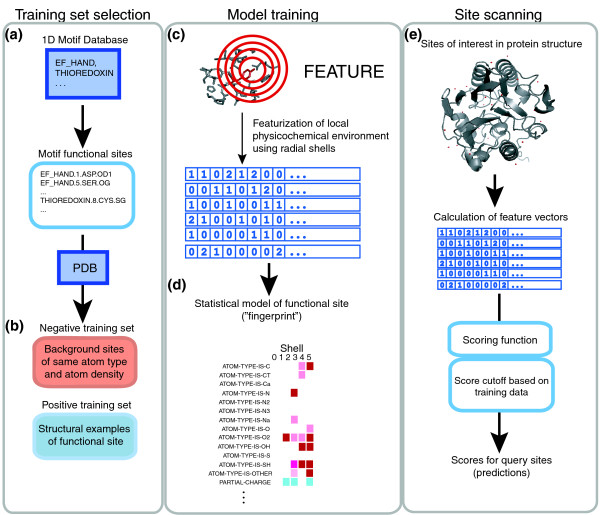
Overview of the SeqFEATURE pipeline. SeqFEATURE forms training sets by **(a) **extracting sequence (one-dimensional) motifs from PROSITE and **(b) **identifying the annotated functional amino acids. We extract examples of the one-dimensional motif with known three-dimensional structure in the PDB and center FEATURE training sites on each functional atom of each functional amino acid annotated in the PROSITE pattern. We choose negative sites matched for atom density randomly from the PDB that do not contain the function. **(c) **FEATURE then creates a model of the sites by summarizing the biochemical and biophysical features found in concentric shells around the functional atom center. **(d) **The resulting three-dimensional fingerprint specifies the properties that are in relative abundance or paucity in the site, representing the model. **(e) **A protein of interest is converted into features and scored with the model using a naïve Bayes scoring function, and predictions are made using score cutoffs, which can be based on desired performance statistics. The scores are calibrated into Z-scores using the training set used to derive each model.

The functional 'fingerprint' of each model (as shown in Figure 7d) also allows detailed understanding of the physicochemical environment representative of that type of functional site, and detailed inspection of potential positives may boost confidence of positive predictions or help explain the existence of any false positives. Even if the SeqFEATURE prediction is not entirely accurate, the fact that it is based on a representation of the local physical and chemical environment means that we can still make interesting observations about what properties helped the site score highly, and which additional properties may be necessary for the site truly to contain the predicted function.

Most importantly, since SeqFEATURE is not dependent on sequence or overall structural fold, it can be used when either the sequence or the structure is novel. This became evident when we compared the performance of the different methods at low sequence identities and low structural similarities, and found that SeqFEATURE shows a trend to being more sensitive than sequence-based methods at low sequence identities and more sensitive than other structure-based ones at low structural similarities. As shown with the three TargetDB examples, SeqFEATURE is able to predict function where other methods are not. Further inspection of the putative sites reveals compelling evidence for SeqFEATURE's predictions. The ability to provide useful predictions on novel structures will become more and more important as structural genomics matures, and SeqFEATURE demonstrates robust performance in this area.

## Conclusion

Advances in protein structure determination have led to an increase in unannotated structures, many with low sequence identity to known proteins. Our method, SeqFEATURE, uses functional sequence motifs to seed training sets from which three-dimensional models of the function are built. We used SeqFEATURE to construct a large library of three-dimensional functional site models from PROSITE motifs and scanned uncharacterized structural genomics targets from TargetDB for function. SeqFEATURE's descriptive and intuitive models show comparable performance to existing sequence- and structure-based methods. Importantly, SeqFEATURE models retain robust performance when sequence identity and structural similarity are reduced. Methods such as SeqFEATURE that do not rely on strict sequence or structure conservation will be valuable tools for annotating novel protein structures.

## Materials and methods

SeqFEATURE (Figure 7) is a method for automatically selecting training sets and building structural models within the FEATURE framework, a system for modeling functional sites in protein structures that has been published previously [32]. Here, we summarize the FEATURE algorithm and present SeqFEATURE in more detail.

### The FEATURE algorithm

FEATURE builds statistical three-dimensional models of the local environment around a functional site given training sets of positive and negative examples. These models can then be used to evaluate test sites and predict whether they have particular functions. FEATURE calculates a number of physicochemical properties (Table 6) at varying radial distances from the site center and creates a feature vector containing the values of each property in each radial volume (Figure 7c). Both atomic and residue level properties are examined, allowing the functional site to be described at multiple levels. The structural model is constructed by comparing the statistical distribution of properties between positive and negative sites.

**Table 6:** Physicochemical properties used by the FEATURE algorithm

Atom-based	Molecule-based	Residue-based	Secondary structure-based
ATOM-TYPE-IS-C	PARTIAL-CHARGE	RESIDUE_NAME_IS_ALA	SECONDARY_STRUCTURE1_IS_3HELIX
ATOM-TYPE-IS-CT	HYDROXYL	RESIDUE_NAME_IS_ARG	SECONDARY_STRUCTURE1_IS_4HELIX
ATOM-TYPE-IS-Ca	AMIDE	RESIDUE_NAME_IS_ASN	SECONDARY_STRUCTURE1_IS_5HELIX
ATOM-TYPE-IS-N	AMINE	RESIDUE_NAME_IS_ASP	SECONDARY_STRUCTURE1_IS_BRIDGE
ATOM-TYPE-IS-N2	CARBONYL	RESIDUE_NAME_IS_CYS	SECONDARY_STRUCTURE1_IS_STRAND
ATOM-TYPE-IS-N3	RING-SYSTEM	RESIDUE_NAME_IS_GLN	SECONDARY_STRUCTURE1_IS_TURN
ATOM-TYPE-IS-Na	PEPTIDE	RESIDUE_NAME_IS_GLU	SECONDARY_STRUCTURE1_IS_BEND
ATOM-TYPE-IS-O	VDW-VOLUME	RESIDUE_NAME_IS_GLY	SECONDARY_STRUCTURE1_IS_COIL
ATOM-TYPE-IS-O2	CHARGE	RESIDUE_NAME_IS_HIS	SECONDARY_STRUCTURE1_IS_HET
ATOM-TYPE-IS-OH	NEG-CHARGE	RESIDUE_NAME_IS_ILE	SECONDARY_STRUCTURE1_IS_UNKNOWN
ATOM-TYPE-IS-S	POS-CHARGE	RESIDUE_NAME_IS_LEU	SECONDARY_STRUCTURE1_IS_HELIX
ATOM-TYPE-IS-SH	CHARGE-WITH-HIS	RESIDUE_NAME_IS_LYS	SECONDARY_STRUCTURE1_IS_BETA
ATOM-TYPE-IS-OTHER	HYDROPHOBICITY	RESIDUE_NAME_IS_MET	SECONDARY_STRUCTURE1_IS_COIL
ATOM-NAME-IS-ANY	MOBILITY	RESIDUE_NAME_IS_PHE	SECONDARY_STRUCTURE1_IS_HET
ATOM-NAME-IS-C	SOLVENT-ACCESSIBILITY	RESIDUE_NAME_IS_PRO	SECONDARY_STRUCTURE1_IS_UNKNOWN
ATOM-NAME-IS-N		RESIDUE_NAME_IS_SER	
ATOM-NAME-IS-O		RESIDUE_NAME_IS_THR	
ATOM-NAME-IS-S		RESIDUE_NAME_IS_TRP	
ATOM-NAME-IS-OTHER		RESIDUE_NAME_IS_TYR	
		RESIDUE_NAME_IS_VAL	
		RESIDUE_NAME_IS_HOH	
		RESIDUE_NAME_IS_OTHER	
		CLASS1_IS_HYDROPHOBIC	
		CLASS1_IS_CHARGED	
		CLASS1_IS_POLAR	
		CLASS1_IS_UNKNOWN	
		CLASS2_IS_NONPOLAR	
		CLASS2_IS_POLAR	
		CLASS2_IS_BASIC	
		CLASS2_IS_ACIDIC	
		CLASS2_IS_UNKNOWN	

These properties are expressed at the atomic, molecular, residue and secondary structural levels of abstraction. The properties at the atomic, molecule and secondary structural level are designed to make the FEATURE models relatively less dependent on primary amino acid sequence, in an attempt to improve performance on highly divergent (or convergent) sites. Most of these properties are simply counts of the property, and a few are continuous valued, as discussed in [32].

In a model for a particular functional site, properties are described as either significantly more present, more absent, or having no significant difference in the positive sites compared to negative sites using the Wilcoxan rank sum test [32]. The significant properties can be visually displayed as a color-coded matrix unique to that model, which we call its fingerprint (Figure 7d).

Using a naïve Bayes scoring function, FEATURE can then evaluate the likelihood that a query site contains the function described by a particular model. A feature vector is created for the query site in the same way as for the training sites, and a likelihood score is calculated assuming independence of each individual feature *υ*_*i*_:

$$\text{Score}=\sum_{i}\log\left[\frac{P(site|\upsilon_{i})}{P(site)}\right]$$

A score cutoff for classifying a query site can be chosen for each model according to the user's desired performance criteria (Figure 7e).

### SeqFEATURE

#### Training set selection

SeqFEATURE adds to the FEATURE framework by using one-dimensional sequence motifs as seeds for generating training sets of structural examples (Figure 7a). This method was first introduced by Liang *et al*. [34] in a single application to calcium binding by EF-hand motifs, and is extended and applied here into a full library of functional site models. To build the library of models, we extracted structural examples of PROSITE functional site patterns from the ASTRAL40 compendium [38], which is a nonredundant subset of protein domains in the PDB. We required training sets to have a minimum of five structural examples.

PROSITE patterns are regular expressions that specify the amino acids permitted at each position of the motif. We defined functional site centers to be the functional atom(s) of annotated functional residues in each pattern, for example, the gamma oxygen of serine, or SER.OG. For patterns with multiple functional residues or multiple functional atoms, we built multiple models for the same PROSITE pattern. For example, the PROSITE pattern EGF_1 has functional cysteine residues at positions 1, 3, and 7, so there are three models centered at three atoms in this pattern - EGF_1.1.CYS.SG, EGF_1.3.CYS.SG, and EGF_1.7.CYS.SG. Models derived from PROSITE are always named using a four-part naming scheme specifying the motif, the position in the motif, the residue at that position, and the atom within that residue upon which the model is centered. See Table 1 for a complete list of SeqFEATURE models.

Positive training sets consist of PDB coordinates of functional atoms as described above, extracted from structures containing that particular pattern. We selected negative training sets randomly from identical residues in the rest of the PDB whose atom compositions and densities are similar to the positive sites. In order to define the background distribution of the functional site environments, we used a thousand times as many negative sites as positive sites for each model, when possible, but never less than 4,000.

#### Model cross-validation and evaluation

We internally evaluated each model using five-fold cross validation by partitioning the positive and negative training sets randomly into five blocks. For each run, we used four blocks to build the model and tested performance on the remaining block. To compare results across runs, we transformed the scores into Z-scores by standardizing to the mean and standard deviation of the negative score distribution.

To measure performance, we employ ROC curves, which plot the true positive rate (sensitivity, or the ratio of true positive predictions to all true positives) against the false positive rate (1-specificity, or the ratio of false positive predictions to all true positives) at varying Z-score cutoffs. We also use PPV versus sensitivity to gauge the performance of a model. Sensitivity, specificity, and PPV are calculated as follows:

$$\text{Sensitivity}=\frac{\text{\# of true positive predictions}}{\text{total \# of true positives}}$$

$$\text{Specificity}=\frac{\text{\# of true negative predictions}}{\text{total \# of true negatives}}$$

$$\text{Positive predictive value}=\frac{\text{\# of true positive predictions}}{\text{total \# of positive predictions}}$$

The AUC estimates the probability that a random positive site will be scored higher than a random negative site, and provides a summary measure of the performance of the model. The final models used all of the training examples, and include score cutoffs calculated for 95%, 99%, and 100% specificity based on cross validation data.

### Comparison to other function prediction methods

The manually curated PROSITE record for each pattern contains known true positives, false positives, and false negatives predicted by that pattern, listed using Swiss-Prot identifiers. We treated each Swiss-Prot ID as a unique protein. Taking existing mappings between Swiss-Prot and the PDB, we also converted each list into a list of corresponding PDB structures to use as input to SeqFEATURE and other structure-based prediction methods. Thus, our positive test set consisted of Swiss-Prot IDs and PDB structures for proteins annotated as true positives and false negatives in PROSITE, and our negative test set consisted of Swiss-Prot IDs and PDB structures for proteins annotated as false positives. We removed all positive training set structures from the test sets and filtered the test structures to ensure that they contained the functional regions described by the appropriate PROSITE pattern.

Using these test sets, we compared performance among PROSITE, Pfam, Gene3D, HMMPanther, SSM, 3D templates (reverse template type), and SeqFEATURE. In order to ensure consistency across the comparisons, we restricted the analysis to patterns that had at least one model with an AUC >0.75 and that also mapped unambiguously to the pattern database or tool being compared. For example, to determine the Pfam assignment for a particular pattern, we looked up the set of Pfam assignments for each structure in the training set using Pfam's publicly available mappings. Unambiguous assignments were those for which either 100% of the training set mapped to the same Pfam family, or for which the Pfam family clearly matched the PROSITE pattern (for example, PROSITE pattern GLYCOSYL_HYDROL_F10 and Pfam family 'Glyco_hydro_10'). Forty-two PROSITE motifs had both an AUC >0.75 and a positive test set independent of the training set (TRYPSIN_HIS was excluded due to it being nearly identical to TRYPSIN_SER), and, of these, 31 mapped unambiguously to Pfam, 12 to Panther, and 29 to Gene3D.

Because structure-based methods such as 3D templates and SSM are more computationally expensive to run than SeqFEATURE and the sequence-based methods, we split the comparison into two parts. The first part compared PROSITE, Pfam, HMMPanther, Gene3D, and SeqFEATURE, and covered the unambiguous portions of the test sets in their entirety. PROSITE's predictions came directly from its annotations. For the other sequence-based methods, we analyzed the test set proteins using each tool and marked a protein as a positive prediction if at least one of its mapped predictions matched the unambiguous assignment for the pattern being tested. HMMPanther and Gene3D were run from the InterPro servers using the stand-alone downloadable Perl client [39]. Pfam's predictions were taken directly from their publicly available mapping file. For SeqFEATURE, we classified a protein as positive if at least one of its mapped PDB structures scored above the specified cutoff for at least one model derived from that pattern. Since SeqFEATURE cutoffs are variable, we tested performance at 95%, 99%, and 100% specificity cutoffs.

To compare SSM, 3D templates, and SeqFEATURE, we limited our test sites to those derived from PROSITE patterns that mapped to EC numbers. Since 3D templates (reverse template type) and SSM both return protein structures rather than a named function as output, we used EC numbers to evaluate predictions made by SSM and 3D templates. We determined the set of EC numbers corresponding to each pattern's training set and randomly sampled 29 positive sites and 15 negative sites from the EC-compatible subset of test sites. We then took the top prediction below 95% sequence identity to the query for each test site from SSM and 3D templates that had an EC number, and considered it a positive prediction if the EC number matched any of the EC numbers assigned to the relevant PROSITE pattern. We determined SeqFEATURE predictions by evaluating whether each structure scored above the 95%, 99%, and 100% cutoffs for at least one model derived from the appropriate pattern.

Importantly, we compared the sequence-based methods to SeqFEATURE using low sequence identity test sets. We computed all pairwise sequence alignments between structures in the positive test set and the training set for each pattern using Jaligner, a freely available Smith-Waterman alignment software package [40], and constructed a new test set consisting of those test structures that had less than 35% sequence identity to structures in their corresponding training set. This comprised the low sequence identity positive test set, which we analyzed according to sequence identity thresholds differing by 2% (<35%, <33%, and so on, down to <25%). We looked up the predictions from the sequence-based methods for the low sequence identity test set at each of these thresholds.

From the low sequence identity test set, we conducted pairwise structural similarity searches between each structure and the structures in the corresponding training sets using DALI, a publicly available tool for calculating structural similarity [41]. We discarded any structure that matched a training set structure with a Dali Z-score greater than 10.0. The remaining structures all had no significant matches, or only low-confidence matches, to their positive training sets. We looked up the predictions from 3D templates, SSM, and SeqFEATURE (at the three different cutoffs) for the low structural similarity test set.

### Protein Data Bank scan

Any PDB structure can be scanned with any SeqFEATURE model to generate a list of predictions. The March 2006 version of the PDB contains about 35,600 structures, about 95% of which are proteins. We extracted lists of each of the relevant potential functional atoms from each protein structure (ARG.NE, ASP.OD1, ASP.OD2, CYS.SG, and so on), including all chains. This resulted in 90,919,770 potential sites. We then scored all of these sites with the corresponding models that were built on that particular type of functional atom. The entire scan (extracting and scoring) took about one day to complete on fourteen parallel processors. To analyze the scan data, we filtered out redundant scores from proteins with multiple, identical chains.

### TargetDB prediction analysis

We focused our scan analysis on structures listed in TargetDB, the database for targets from structural genomics centers [4]. Using the headers of released PDB files, we filtered for those that lacked functional annotation; for example, 'STRUCTURAL GENOMICS,' 'UNKNOWN FUNCTION', 'HYPOTHETICAL PROTEIN', and so on. We scanned these structures with the entire library of SeqFEATURE models and manually examined the predictions for those hits that satisfied the following two conditions: the prediction was for a model that has an AUC >0.85; and the hit scored above the 100% specificity cutoff or well within the positive Z-score distribution for that model. We then compared each prediction to the results of PROSITE, Pfam, HMMPanther, Gene3D, SSM, and 3D template searches on those structures, and prioritized cases where the sequence-based methods produced no significant predictions.

### WebFEATURE function prediction server

All of the models may be used to scan any protein structure on WebFEATURE, our web-accessible function prediction server [35]. Results from the PDB scan are also available for download. Source code for FEATURE is available from SimTK [42].

## Abbreviations

AUC, area under the curve; EC, Enzyme Commission; GO, Gene Ontology; HMM, Hidden Markov Model; PDB, Protein Data Bank; PPV, positive predictive value; ROC, receiver operating characteristic; SCOP, Structural Classification of Proteins; SSM, Secondary Structure Matching.

## Authors' contributions

SW performed the model training, performance evaluation, PDB analysis, case study analysis, and wrote the manuscript. MPL conceived and preliminarily carried out model training and performance evaluation. RBA conceived and directed the study and helped write the manuscript.
